# Toward pulping process for enhancing the RS-black liquors as precursor of activated carbons for aqueous adsorbent purposes

**DOI:** 10.1038/s41598-023-47447-4

**Published:** 2023-11-16

**Authors:** Vivian F. Lotfy, Zhichao Bao, Xuesong Zhou, Altaf H. Basta, Shiyu Fu

**Affiliations:** 1 Cellulose and Paper Department, National Research Centre, El Buhouth St., Dokki, 12622 Giza Egypt; 2 State State Key Laboratory of Pulp and Paper Engineering, South China University of Technology, Guangzhou, 510640 China

**Keywords:** Materials science, Other nanotechnology

## Abstract

This work deals with providing a green pulping process of rice straw with zero waste discharged, via valorization of its by-product as a promising precursor for production of carbon nanostructures. The carbon nanostructures (BL-CNSs) from rice straw pulping liquors (BLs) are prepared in one step with phosphoric acid activation. The carbon nanostructures (BL-CNSs) from rice straw pulping liquors (BLs) are prepared in one step with phosphoric acid activation. The optimal pulping approach for achieving effective adsorbent (BL-CNSs) of cationic and anionic dyes is recommended from using different BLs precursors resulting from different reagents (alkaline, neutral, and acidic reagents). The carbon precursors are characterized by elemental, thermal (TGA and DTG) and ATR FTIR analyses. While the impact of pulping route on performance of CNSs is evaluated by their adsorption of iodine, cationic dye and anionic dye, as well as ATR-FTIR, textural characterization, and SEM. The data of elemental analysis displayed a high Carbon content ranges from 57.85 to 66.69% suitable for CNSs preparation, while the TGA showed that Sulphur-containing BLs (Kraft, neutral sulfite and acidic sulfite) have higher degradation temperature and activation energies as compared with other BLs. The optimum BL-CNSs adsorbent is prepared from the disposed neutral sulfite black liquor, with the following characteristics: cationic dye adsorption capacity 163.9 mg/g, iodine value 336.9 mg/g and S_BET_ 310.6 m^2^/g. While the Kraft-CNSs provided highest anionic adsorption (70.52 mg/g). The studies of equilibrium and kinetic adsorption of dyes showed that the adsorption equilibrium of all investigated BL-CNSs toward MB follow the Langmuir and mainly Freundlich models for BB adoption. Their adsorption kinetics are a good fit with the pseudo-second-order model. The textural characterization and SEM revealed the CNSs exhibit a mixture of mesoporous and microporous structure.

## Introduction

In recent decades, carbon nanostructure materials have become attractive due to their unique properties, such as their high specific surface area, high mechanical strength, optical transparency, high electrical conductivity, and great thermal stability^1,2^. These extraordinary properties can be introduced in a variety of forms, for example, powders, aerogels, fibres, composites, tubes, sheets, monoliths, etc.^3–6^. Carbon-based nanomaterials have tremendous potential for environmental applications as powerful adsorbents due to their high porosity. Moreover, it has been widely investigated for biomedical (tissue engineering, bio-imaging, drug, and gene delivery), optical materials, and energy storage (electrodes for supercapacitors, hydrogen storage systems, Li-ion batteries, fuel cells, and solar cells) applications^7–10^. Conventionally, carbon nanomaterials are made from graphene, coal, or charcoal. However, the renewable resources (animal and plant-derived biomasses that are carbon-rich) are more intriguing due to their sustainability. Agricultural waste (such as rice by-products, bamboo, bagasse, banana peel, etc.), shells, scales, and chitin are some of the most significant environmentally friendly sources of carbon precursors^11–14^.

Environmentally, the high rate of urbanization and industrialization has caused genuine ecological issues. One of the problems facing the agro-waste paper pulping industry is the discharge of black liquor (BL) by-products^15^. The conventional scenario of BL utilization is based on concentration and combustion in the recovery boiler to generate energy and recover the pulping chemicals. Cracking and corrosion problems in the BL recovery boilers are the most frequent causes for pressure boundary maintenance inspection and replacement^16^. Many recent studies have used several strategies, such as thermochemical, biological, and chemical routes, for the bioconversion of BL into biohydrogen, bioplastic, biodisel, biogas, and chemicals (succinic acid, diethyl ether, and methanol)^17^. Also, BL or BL-lignin can be used for wood preservatives, adhesives and paint manufacture, control release systems for agriculture, dispersing and stabilizing agents for dyes, and carbon precursors^18–20^.

The objective of this study is the utilization of different rice straw pulping black liquors as carbon precursors for the production of carbon nanostructures using phosphoric acid chemical activation. The role of pulping agents of rice straw (sodium hydroxide, sodium hydroxide-additives, potassium hydroxide/ammonium hydroxide, Kraft, neutral sulfite, and sulfite) on the adsorption process of resulted BL-CNSs toward cationic and anionic dyes was evaluated. The characterization of carbon precursors is carried out by elemental analysis, FTIR, and thermal analyses. While the performance of BLs-based carbon nanostructures is assessed from their cationic and anionic adsorption behavior, FTIR, texture properties, and SEM) in comparison with previously carbon-based nanomaterials.

## Experimental

### Chemicals and materials

All the chemical reagents are analytically pure. The sources of reagents and materials were described in detail, as follows:

- Black liquor precursors: Egyptian agriculture waste (rice straw) (RS) was used as agrofibers for the pulping process to get the black liquors (BLs) that represent carbon precursors. The pulping reagents used for the pulping process were sodium hydroxide, potassium hydroxide, sodium sulfite, sodium carbonate, and anthraquinone (AQ), which were purchased from El-Nasr Pharmaceutical Chemical Co. (ADWIC). Sodium sulphide and sodium borohydride (BH) were supplied from Riedel-de Haën AG in Seelze Hannover (Germany), while ammonium hydroxide (25%) was supplied from BDH Limited (Germany).
- For activation, phosphoric acid (85% was supplied from El-Nasr Pharmaceutical Chemical Co. (ADWIC).
- The adsorbate for estimating the adsorption efficiency of carbon-based nanomaterials: methylene blue dye (MB), Coomassie brilliant blue R-250, and iodine were purchased from Alfa Chemicals Co., SD Fine-chem Ltd. (India), and El Nasr Pharmaceutical Chemical Co., respectively.

### Preparation and proximate analysis of carbon nanostructure precursors (black liquors)

Pulping of rice straw (RS) was carried out using different reagents such as soda [with anthraquinone (AQ), sodium borohydride (BH), or their mixture as additives], potassium hydroxide/ammonium hydroxide, Kraft, neutral sulfite, and sulfite pulping conditions. Pulping processes were conducted under the following operating conditions: in autoclaves with a liquor ratio of 6:1 for 1 hour at 120 °C. 18.6% Na_2_O equivalent for NaOH pulping without and with 1% of anthraquinone, 3% sodium borohydride, or both of them were prepared. The black liquor obtained from soda pulping was labelled: BL1 for NaOH pulping, BL2 for NaOH–1%AQ pulping, BL3 for NaOH–3%BH pulping, and BL4 for NaOH–1%AQ–3%BH pulping. The black liquor of the mechanically treated RS before pulping with NaOH–1%AQ–3%BH was labelled BL5. The BLs resulted from a potassium hydroxide/ammonium hydroxide mixture (ratio 1:5) of the Kraft process (10% active alkali and 25% sulfidity), neutral sulfite pulping (a mixture of sodium sulfite and sodium carbonate with a mass ratio of 4:1), and acidic pulping using sodium sulfite, labelled BL6, BL7, BL8, and BL9, respectively.

### Characterization of carbon precursors (BLs)

#### Elemental analysis

The main elements (C, N, H, S, and O) of the resulting BLs were determined using Profilenc Technologies ECS 8020 CHNS-O (Italy) as an elemental analyzer based on the Dumas combustion method**.** The analyzer represents an evolution of elemental analysis techniques based on sample combustion and chromatographic separation.

#### Thermo-gravimetric analyses

Thermo-gravimetric analysis (TGA and DTG) of the BLs as precursors for CNS preparation are carried out using Setaram LABSYS EVO STA, France. The analysis is performed over a temperature range of 30–600 °C using a heating scan rate of 10 °C/min in an inert atmosphere of nitrogen gas (30 mL/min). The Kinetic parameters of thermal degradation were calculated according to references^21–24^.

#### ATR-FTIR spectroscopy

The prepared black liquors were subjected to FTIR analysis using a VERTEX 80v-FTIR spectrophotometer, Germany. The spectral data was collected in the range 400–4000 cm^–1^ to assign the function groups of the prepared samples.

### Preparation of carbon nanostructures from BL precursors (BL-CNSs)

The activation of dried BLs was carried out using phosphoric acid as a chemical activator in a ratio of 3:1 (phosphoric acid: dried BLs) and pyrolyzed in a horizontal tubular furnace at 450 °C for 60 min (Kouotou et al., 2013) in the absence of air.

### Characterization of carbon nanostructures (BL-CNSs)

#### Adsorption studies

In order to evaluate the adsorption behavior of BL-CNSs in aqueous solutions, their adsorption to iodine, methylene blue (MB) (as an example of a cationic dye) and brilliant blue (BB) (as an example of an anionic dye) was estimated.

##### Iodine value

The iodine number and methylene blue dye adsorption studies were carried out at room temperature (25 ℃). The iodine number test is considered to be the most basic parameter to characterize the micro-porosity developed in carbon-based materials. The Iodine value experiment was determined according to the procedure established by ASTM, 2006^25^.

##### Batch equilibrium studies

Adsorption experiments were carried out in batch mode. A stock solution of methylene blue (MB) and brilliant blue (BB) dyes was prepared. For each experiment, 10 mL of a known dye solution was added to 25 mg of CNSs. The mixture was kept at 30 °C and agitated at a constant speed (100 rpm). After 24 and 48 h, the dye solutions were separated from the adsorbent by filtration. The dye concentration in the filtered solutions was analyzed using a UV–Vis Single-Beam spectrophotometer (UV1720, USA). The absorbances at 664 nm and 553 nm were used to calculate the equilibrium adsorption of the MB and BB dyes^26^. The MB and BB adsorption capacities at equilibrium (q_e_, mg/g) were calculated using the Langmuir, Freundlich, Temkin, and Dubinin-Radushkevich (D-R) isotherms^27–30^, as follows:

$${Q}_{e}=\frac{\left({C}_{o}-{C}_{e}\right)\mathrm{V}}{\mathrm{W}}$$

where C_o_ and C_e_ (mg/L) are the liquid-phase concentrations of the dye at initial and equilibrium. V (L) is the volume of the dye solution. W (g) is the weight of CNSs.

**Table:** 

Batch model	Equation form	Ref
Langmuir isotherm	$\frac{{C}_{e}}{{q}_{e}}=\frac{1}{\mathrm{b}{q}_{m}}+\frac{{C}_{e}}{{q}_{m}}$	^27^
Freundlich isotherm	${\mathrm{log}qe=\mathrm{log}K}_{F}+\frac{1}{n} log {C}_{e}$	^28^
Temkin isotherm	${q}_{e}= \frac{RT}{B}\mathrm{Ln }{\mathrm{A}}_{T} +\frac{RT}{B}\mathrm{Ln }{\mathrm{C}}_{T}$	^29^
D-R isotherm	$Ln {q}_{e}=Ln {q}_{m}-\beta {\varepsilon }^{2}$ $\varepsilon =RT Ln (1+\frac{1}{{C}_{e}})$ ${E}_{DR}=\frac{1}{\sqrt{-2\beta }}$	^30^

##### Adsorption kinetic studies

The kinetic adsorption of the investigated dyes (MB and BB) on BL-CNSs can be studied by applying the Lagergren first-order, pseudo-second order, and intraparticle diffusion models at 500 and 50 mg/L, respectively, with time intervals ranging from 1 to 48 h. The rate equations that have been most widely used for the adsorption of an adsorbate from an aqueous solution, are expressed by the equations^31–33^.

**Table:** 

Kinetic model	Linear form	Plots	Ref
Lagergren first order	$\mathrm{ln}\left({\mathrm{q}}_{\mathrm{e}}-{\mathrm{q}}_{\mathrm{t}}\right)={\mathrm{lnq}}_{\mathrm{e}}-{\mathrm{K}}_{1}$	$\mathrm{ln}\left({q}_{e}-{q}_{t}\right)$ versus t	^31^
Pseudo-second order	$\frac{\mathrm{t}}{{\mathrm{q}}_{\mathrm{t}}}= \left[\frac{1}{{\mathrm{K}}_{2}{\mathrm{q}}_{\mathrm{e}}^{2}}\right]+\frac{1}{{q}_{e}} t$	t/q_e_ versus t	^32^
Intraparticle diffusion	${\mathrm{q}}_{\mathrm{t}}= {\mathrm{K}}_{\mathrm{id }}{\mathrm{ t}}^\frac{1}{2}+\mathrm{C}$	q_t_ versus t^1/2^	^33^

Where q_e_ and q_t_ are the amount of dye adsorbed per unit mass of the adsorbent (in mg g^−1^) at equilibrium time and time t, respectively, k is the rate constant, and C is the intraparticle diffusion constant.

Based on the foregoing adsorption and thermal stability data, some BL-CNS samples were selected for further examination, e.g., ART-FTIR, Textural characterization and SEM.

#### FTIR-ATR analysis

The prepared BL-CNSs were subjected to FTIR analysis using a VERTEX 80 v-FTIR spectrophotometer from Germany. The spectral data was collected in the range 400–4000 cm^−1^ to assign the function groups of the prepared samples.

#### Textural characterization

Textural characterization of CNS samples prepared from BLs was carried out by nitrogen adsorption–desorption isotherms that performed 77K using the BELSORP III analyzer series in Japan. The samples were degassed in an oven at 250 °C for 24 h and analyzed using BELMaster version 7.3.1 software. The instrument was adjusted to a vacuum degree before the measurement reached 5.267E−5 Pa. This test was performed to get the surface area and pore volume using the BET, BJH, and t-plot methods.

#### Scanning electron microscope (SEM)

The morphology of the investigated BL-CNSs was examined by scanning electron microscopy (SEM). The samples were examined using a Quanta 3D 200i-Russia system running at 30 kV.

## Results and discussion

### Characterization of carbon precursors (BLs)

#### Elemental analysis

The elemental analysis of dried black liquors (as carbon precursors) is presented in Table 1. In general, these elements correspond to the content of lignin and other low-molecular compounds found in black liquor. The carbon, oxygen and hydrogen contents of BLs range from 57.85 to 66.69%, from 15.2 to 21.2% and 4.05–5.11%, respectively. The highest carbon content (greater than 60%) corresponds to BL1–BL5, which are related to the NaOH or NaOH-additives pulping reagents. The BL1 and BL5 have the greatest lignin removal (34.8% and 39.4%) from RS pulping, respectively and resulting in high carbon contents of 65.27 and 66.69%, respectively. For other pulping reagents, such as Kraft-BL (BL7) the maximum lignin and silica removal reached 73.11% and 64.82%, which leads to moderately distributed carbon at 58.9%, together with highest oxygen content (21.2%). With respect to the sulphur content (4.95–5.5%), they are characterized to BL samples that resulted from pulping by sulfur-containing reagents (Kraft, neutral sulfite, and sulfite pulping; BL7–BL9); while nitrogen-containing BL (12.5%) is produced from using KOH/NH_4_OH pulping reagents (BL6). From the foregoing data, the BLs with high carbon content are convenient as carbon precursors for carbon nanostructure preparation.

**Table 1:** Pulping condition, CHNS-O elemental analysis in different black liquors and the removed lignin and silica from RS in BLs.

Conditions of RS-pulping	Code	Elemental analyses							Removed lignin and silica in BLs		
		C, %	H, %	N, %	S, %	O, %	H/C	O/C	Lignin removal, %	Silica removal, %	CNSs yield, %
NaOH (18.6% Na_2_O)	BL1	66.69	4.05	–	–	19.7	0.061	0.295	34.86	57.96	55.58
NaOH (18.6% Na_2_O)-1%AQ	BL2	65.33	4.11	–	–	15.2	0.063	0.233	26.96	48.41	63.57
NaOH (18.6% Na_2_O)-3%BH	BL3	63.75	4.08	–	–	16.3	0.064	0.256	13.32	41.6	54.39
NaOH (18.6% Na_2_O)-1%AQ-3%BH	BL4	66.31	4.44	–	–	18.5	0.067	0.279	27.66	48.84	65.05
M-NaOH (18.6% Na_2_O)-1%AQ-3%BH	BL5	65.27	4.37	–	–	17.3	0.067	0.265	39.42	58.09	64.95
KOH/NH_4_OH (1:5)	BL6	57.85	5.11	12.51	–	20.32	0.088	0.351	17.81	15.42	38.95
NaOH-Na_2_S (10% active alkali and 25% sulfidity)	BL7	58.9	5.1	–	4.95	21.2	0.087	0.360	73.11	64.82	17.16
Neutral sulfite (Na_2_SO_3_/Na_2_CO_3_ [4:1])	BL8	60.83	4.89	–	5.5	16.7	0.080	0.275	10.83	14.6	17.15
acidic sulfite (Na_2_SO_3_) (18.6% Na_2_O)	BL9	59.88	4.91	–	5.06	20.13	0.082	0.336	18.27	13.78	19.26

According to Pereira, et al*.*^34^, the elemental composition is important for the characterization of fuel because the energy generated by thermal degradation is associated with the enthalpy of carbon, hydrogen, and sulfur. Therefore, low H/C and O/C ratios are desirable for the use of lignocellulosic materials or their byproducts for energy. The ratio of hydrogen to carbon element (H/C ratio) of BL is nearly unaffected by BH and/or AQ added to NaOH pulping reagent (~ 6.2–6.9 × 10^–2^). While the increase in this value was noticed when using other BLs (BL6–BL9), with greatest value for KBL (BL7; 8.9 × 10^–2^) indicates the lignin structure in black liquor, where the highest delignification percentage occurred (73.1%). For the O/C ratio of BL6, BL7 and BL9 RS-pulp is higher than that of other BLs (~ 0.36). The foregoing observation emphasized both delignification degree and silica content are affected on H/C and O/C ratios.

#### Thermo-gravimetric analyses

**Figure 1:**
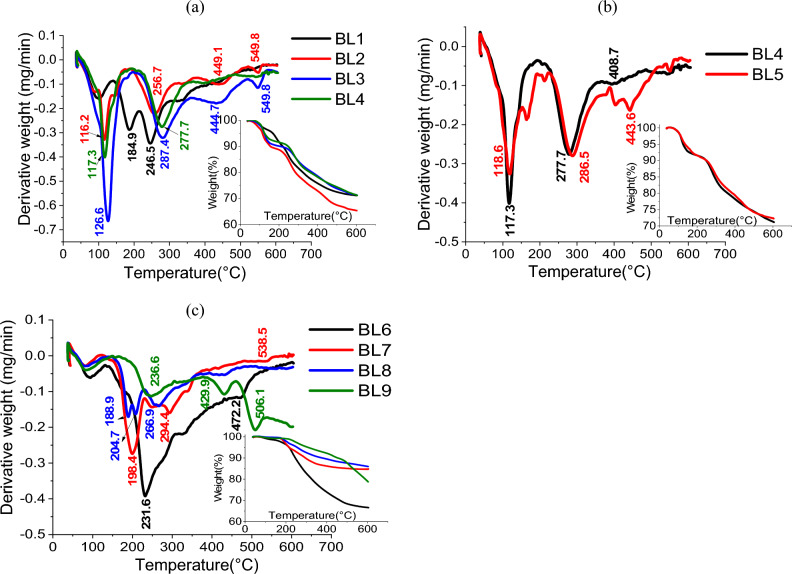
TGA/DTG of BLs from (**a**) soda RS-pulping with additives effect, (**b**) role of mechanical treatment of RS with soda pulping-additives, (**c**) different pulping agents (KOH/NH_4_OH, NaOH-Na_2_S, Na_2_SO_3_/Na_2_CO_3_, Na_2_SO_3_).

TGA-DTG measurements are carried out to predict the thermal behavior of the black liquors during the carbonization processes. From the TGA thermograms (Fig. 1a–c and Tables 2, 3) the degradation of BLs at temperatures less than 130 °C is related to the evaporation of their moisture content and binding water. The decomposition of low-molecular-weight organic components found in BLs occurs at about 150–350 °C (volatilization stage); high-molecular-weight components and polymerized molecules are gradually decomposed at 400–600 °C (carbonization stage). Based on the obtained TGA result, BL samples can be activated and carbonized at 450 °C, making them candidates for our investigation.

**Table 2:** TGA kinetic parameters of BLs from NaOH in absence and presence of additives.

Sample code		Temp. range	DTG peak	Wt. remain % at 450 ℃	n order	R	Se	Ea
BL1	1st	63.7–141.8	98.8	73.78	–	–	–	–
	2nd	142.2–219.3	184.9		2	0.961	0.2	173.153
	3rd	217.8–290.4	246.5		2	0.948	0.215	227.784
								400.937
BL2	1st	57.4–172.2	116.2	69.72	–	–	–	–
	2nd	188.8–323.0	256.8		2	0.97	0.2	153.085
	3rd	380.1–511.3	449.1		2	0.949	0.222	246.772
	4th	522.1–583.7	549.8		1.5	0.961	0.093	532.668
								932.525
BL3	1st	501–192.8	126.6	75.85	–	–	–	–
	2nd	206.5–356.5	278.4		2	0.9707	0.193	145.227
	3rd	356.4–506.6	444.6		2	0.942	0.231	196.806
	4th	521.8–576.3	549.8		2	0.951	0.206	718.612
								1060.645
BL4	1st	69.2–174.4	117.3	75.97	–	–	–	–
	2nd	215.8–352.1	277.7		2	0.97	0.201	168.520
	3rd	364.8–485.2	408.7		2	0.932	0.23	260.693
								429.213
BL5	1st	86.0–152.7	118.5	76.37	–	–	–	–
	2nd	223.5–366.6	286.5		2	0.969	0.193	158.661
	3rd	425.0–485.1	443.6		1.5	0.931	0.196	410.382
								569.042

**Table 3:** TGA kinetic parameters of BLs from other pulping agents rather than NaOH.

Sample code		Temp. range	DTG peak	Wt. remain % at 450℃	n order	R	Se	Ea
BL5	1st	86.0–152.7	118.5	76.37	–	–	–	–
	2nd	223.5–366.6	286.5		2	0.969	0.193	158.661
	3rd	425.0–485.1	443.6		1.5	0.931	0.196	410.382
								569.042
BL6	1st	40.8–131.9	87.4	70.79	–	–	–	–
	2nd	141.1–357.5	231.6		2	0.968	0.23	102.468
	3rd	431.0–507.7	472.2		2	0.939	0.219	405.773
								508.241
BL7	1st	42.3–122.9	80.4	85.49	–	–	–	–
	2nd	152.7–229.9	198.4		2	0.972	0.213	227.420
	3rd	271.8–330.0	294.4		2	0.936	0.22	305.936
	4th	517.0–551.2	538.6		2	0.942	0.2	1127.694
								1661.050
BL8	1st	54.7–138.9	78.9	88.37	–	–	–	–
	2nd	163.37–199.6	188.9		2.5	0.968	0.232	465.951
	3rd	199.6–229.4	204.7		1.5	0.939	0.184	361.524
	4th	229.3–308.1	266.9		2	0.945	0.216	217.936
								1045.411
BL9	1st	50.9–148.2	83.3	90.15	–	–	–	–
	2nd	182.1–304.9	236.6		2	0.972	0.194	164.143
	3rd	388.3–459.1	429.9		2	0.942	0.215	388.181
	4th	478.2–537.6	506.1		2	0.942	0.212	574.737
								1127.061

From the DTG peak temperature of the main degradation peaks (Fig. 1a, b), the role of additives (BH and/or AQ) on soda pulping provides BLs with high thermal stability as compared with soda pulping. It’s clear that BL of NaOH-BH pulping of RS (BL3) has a higher peak temperature (287.4 °C) than other BLs from soda or soda-additive pulping (246.5–286.5 °C). Moreover, the activation energy (E_a_) of thermal degradation, which were calculated using the references^22–24^, confirms the high thermal stability of BL from soda-additive pulping over BL from soda pulping. Whereas the E_a_ of BL1 (soda pulping) is 400.9 kJ/mol and the E_a_ of BL2-BL5 (soda-BH and/or AQ additives) is ranged from 429.2 to 1060.6 kJ/mol, with the maximum activation energy corresponding to BL3 (NaOH-AQ-BH reagent). Tomasso et al.^35^, reported that the thermal decomposition of NaBH_4_ began at a higher temperature (475 °C), which may cause the thermal stability enhancement of BL containing borohydride. On comparing the BLs from the remaining pulping conditions (KOH/NH_4_OH, NaOH-Na_2_S, Na_2_SO_3_/Na_2_CO_3_, Na_2_SO_3_), it is found that the Kraft BL (BL7) has a higher DTG peak temperature (294.4 °C) with an E_a_ ~ 1661 kJ/mol, followed by BL9 with a 236.6°C DTG temperature and 1127.1 kJ/mol activation energy and BL8 with a 266.9°C DTG temperature and 1045.4 kJ/mol activation energy. This may be related to thio-lignin and sulfonyl containing the delignified lignin in BL7–BL9. As can be seen, the higher weight remaining at 450 °C in BL7-BL9 (85.5–90.2%) than in other black liquors (BL1-BL6) (69.7–76.4%) is mainly ascribed to inorganic elements found in the BLs (mainly Sulphur and removed silica)^36,37^. This high weight remains causes a decrease in carbon nanostructure yield (17.2–19.3%); Table 1.

#### ATR- FTIR analysis

**Figure 2:**
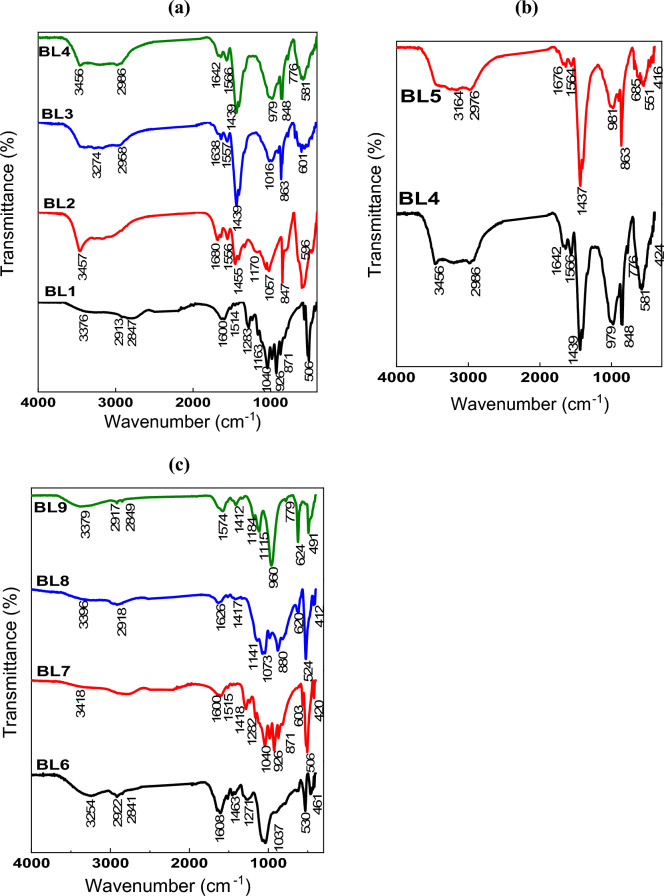
FTIR curves of BLs from (**a**) soda RS-pulping with additives effect, (**b**) role of mechanical treatment of RS with soda pulping-additives, (**c**) different pulping agents (KOH/NH_4_OH, NaOH-Na_2_S, Na_2_SO_3_/Na_2_CO_3_, Na_2_SO_3_).

The dried BLs are analyzed using ATR- FTIR and presented in Fig. 2a–c. As clear from the spectra, all BL samples obtained from the different pulpings exhibit most of the lignin and silica bands. At 3250–3460 cm^–1^, broad bands are assigned to OH stretching vibrations of phenolic lignin, carboxylic acid, or absorbed water. At 2940 cm^–1^, low-intensity bands are assigned to CH asymmetric stretching. The BL lignin is observed with a band at 1650 cm^–1^ and 1550 cm^–1^ for aromatic skeletal vibration (C=C) of lignin (guaiacyl or syringyl). The aromatic ring of the lignin group (stretching and bending of the benzene ring) is also observed in the region of 800–873 cm^–1^ (Esteves et al., 2013). The bands at 1650, 1440, and 1210 cm^–1^ correspond to C=O stretching (non-conjugated ketones or carbonyl groups), CH deformation in CH_3_ or CH_2,_ or phenylpropane skeleton vibrational absorption bands, and ether group C–O–C linkage, respectively. The presence of silica is emphasized by bands in regions 850–1050 cm^–1^ for Si–O–Si asymmetric stretching and 400–800 cm^–1^ for Si–O–Si bending^19,38^.

Figure 2a, b illustrate that CH deformation bands at 1440 cm^–1^ of BLs from soda-additive pulping (BL2-BL5) increased as compared with BL1 (soda pulping), which indicates the role of additives on the skeletal structure of lignin and hydrolyzed hemicellulose. As reported before^39,40^, the hydrolysis of borohydride is carried out in an alkaline solution for hydrogen generation and BO^–^_2_ formation. This explains the great change observed at 1440 cm^–1^ that becomes sharper and more intense for BL3–BL5. The B–O asymmetric stretching of BO_2_ formed during the pulping process between the borohydride in the sodium hydroxide solution at pulping temperature. Moreover, the OH stretching of BL1 appears at 3376 cm^–1^ and shifts to a higher frequency with the pulping additives (BL2–BL5) at about 3450 cm^–1^. Furthermore, the bands related to Si–O–Si stretching or bending become more intense and sharper due to the silica content. Lowering the silica content of BL6, BL8, and BL9 result in a reduction of one of the bands related to Si–O–Si or both, as compared with BL7 or BL1–BL5.

### Characterization of carbon nanostructures (BL-CNSs)

#### Adsorption studies

##### Iodine value

**Figure 3:**
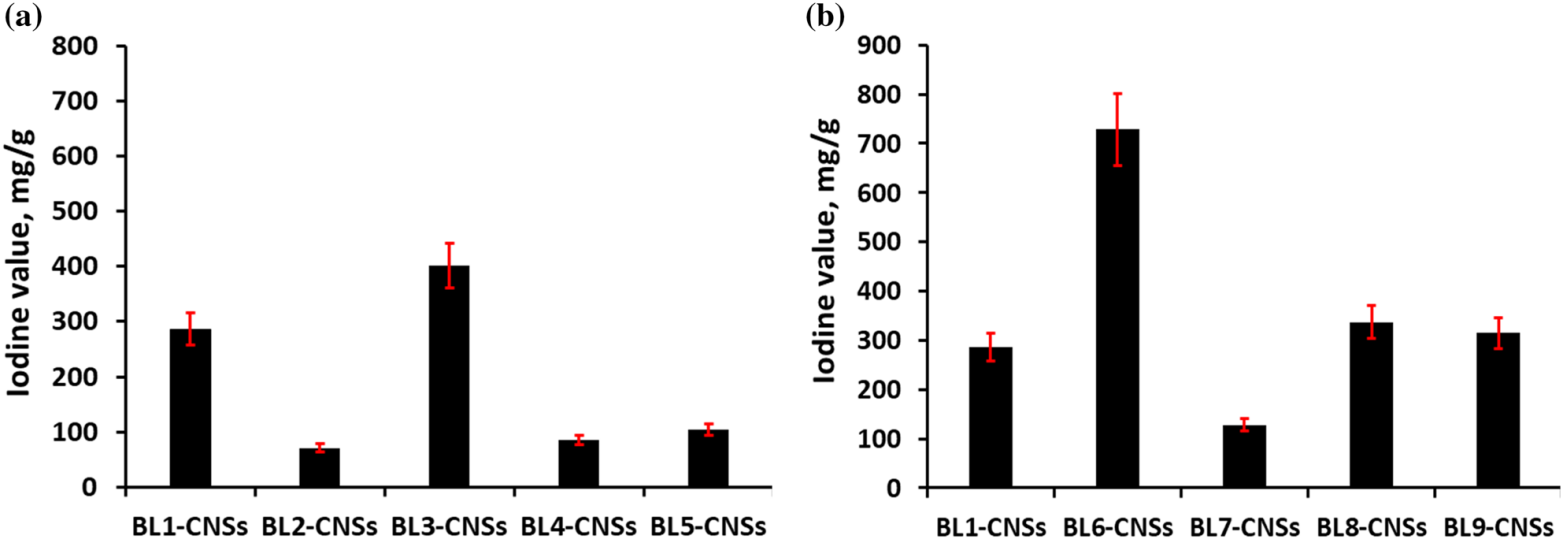
Iodine value of BL-CNSs from (**a**) soda RS-pulping with additives effect and role of mechanical treatment of RS, (**b**) different pulping agents (NaOH, KOH/NH_4_OH, NaOH-Na_2_S, Na_2_SO_3_/Na_2_CO_3_, Na_2_SO_3_).

Figure 3a, b show the iodine adsorption value (mg/g) on the surface of carbon nanostructures resulting from different pulping of RS-black liquors (BL-CNSs) in comparison with the literature. From the results, the iodine value ranges from 71.2 to 728.6 mg/g, with the maximum value of BL6-CNSs obtained from KOH-NH_4_OH black liquor. BL3-CNSs produced from NaOH-BH black liquor also show a high iodine value reaching 401.2 mg/g. For the CNSs from neutral sulfite and acidic pulping-BLs present approximately the same iodine value 336.9 and 314.6 mg/g, respectively. In comparison to the iodine value of our investigation with the literatures, the present results exceeds or similar to many literatures. Mopoung et al.^41^, reported that the iodine adsorption of tamarind seed-based activated carbon with KOH activation ranged from 150 to 300 mg/g; Lim et al.^42^, found the iodine number of oil palm trunk-derived activated carbon was 500–880 mg/g. Saka^43^, studied the production of activated carbon from acorn shell by chemical activation with zinc chloride and reported the iodine number in the range of 37–1209 mg/g. Li et al.^44^, reported that the iodine number of H_3_PO_4_-activated hydrochar at 600–1000 °C temperature was 200–830 mg/g. The foregoing data on iodine values supports the idea that black liquors are efficient precursors for carbon nanostructures with a mainly mesoporous structure.

##### Batch equilibrium studies toward dyes

**Figure 4:**
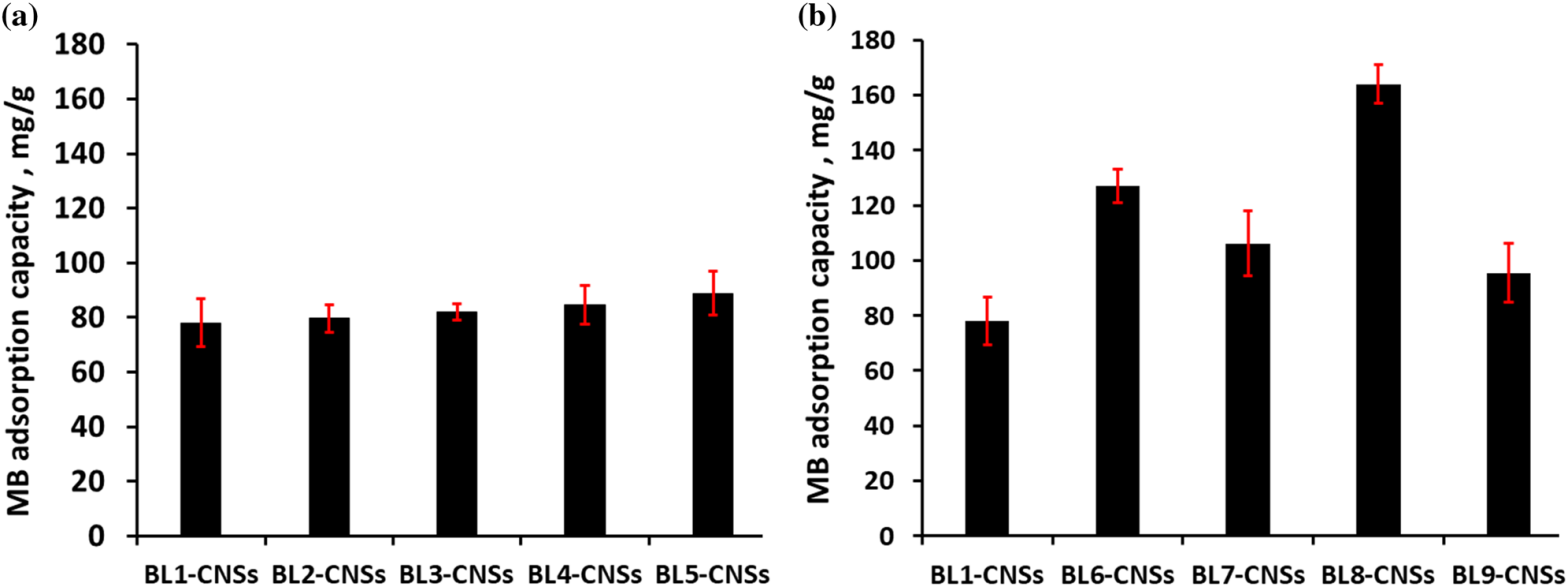
MB adsorption capacity of BL-CNSs from (**a**) soda RS-pulping with additives effect and role of mechanical treatment of RS, (**b**) different pulping agents (NaOH, KOH/NH_4_OH, NaOH-Na_2_S, Na_2_SO_3_/Na_2_CO_3_, Na_2_SO_3_).

**Figure 5:**
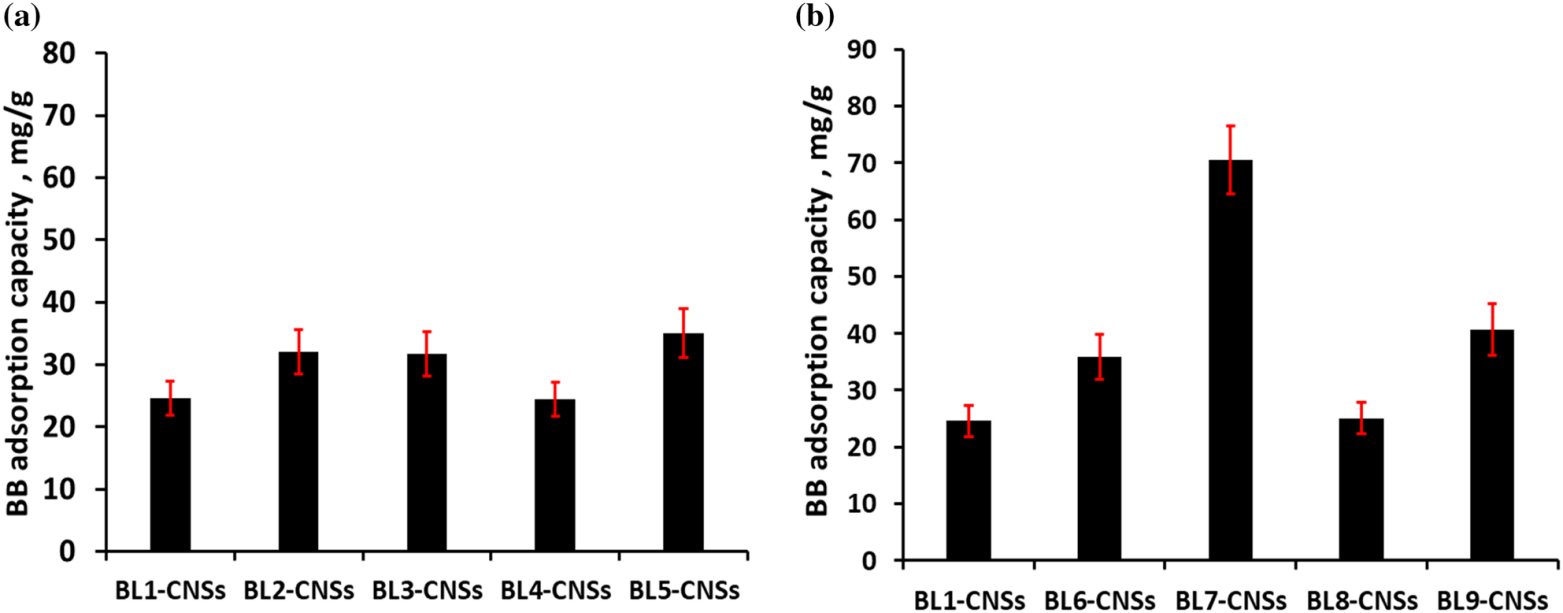
BB adsorption capacity of BL-CNSs from (**a**) soda RS-pulping with additives effect and role of mechanical treatment of RS, (**b**) different pulping agents (NaOH, KOH/NH_4_OH, NaOH-Na_2_S, Na_2_SO_3_/Na_2_CO_3_, Na_2_SO_3_).

The effect of initial concentration of methylene blue (MB) and brilliant blue (BB) dyes on the adsorption capacity of the BL-CNSs is evaluated and the finding results show in Figs. 4 and 5. The resulting data is investigated by Langmuir, Freundlich, Temkin, and Dubinin-Radushkevich (D-R) isotherms as well as the fitting model parameters (Tables 4, 5). As clear, the adsorption capacity of MB (cationic dye) is higher than BB (anionic dye). Due to the semi alkaline pH of MB dye, which makes the surface charge of carbon nanostructure tends to be negatively charged and facilitates the adsorption of cationic dye over the anionic dye. According to Table 4, the equilibrium adsorption of MB dye is described by the Langmuir model with approximately correlation coefficient values (R^2^) greater than 1. With respect to the dimensionless equilibrium parameter (R_L_), it is found between 0 and 1, which means the favored adsorption process. The other isotherm models fit most appropriately in the order Dubinin-Radushkevich (D-R) > Temkin > Freundlich. According to the Langmuir model, the MB adsorption capacity of CNSs form soda additive BLs (BL2-CNSs to BL5-CNSs) is slight increased as compared with BL1-CNSs (Fig. 4a). The maximum adsorption capacity for other BLs-CNSs is observed in case BL8-CNSs (163.9 mg/g) and BL6-CNSs (127.1 mg/g). The efficiency of BLs-CNSs toward MB adsorption is higher than registered in many literatures. The adsorption capacity of MB by bamboo dust or Groundnut shell based activated carbon was 143.2 and 164.9 mg/g^45^, by activated resorcinol–formaldehyde carbon gels was 135 mg/g^46^, by coconut leaves activated carbons was 127–149 mg/g^47^, by almond husk activated carbons was 37.2 mg/g^48^, by bagasse activated carbons was 49.8–56.5 mg/g^49^.

**Table 4:** Langmuir, Freundlich, Temkin and Dubinin–Radushkevich (D–R) isotherm parameters for adsorption of MB dye onto BL-CNSs.

		BL1-CNSS	BL2-CNSS	BL3-CNSS	BL4-CNSS	BL5-CNSS	BL6-CNSS	BL7-CNSS	BL8-CNSS	BL9-CNSS
Langmiur isotherm	Q_m_, mg g^−1^	78.064	79.745	82.169	84.746	88.889	127.065	106.157	163.934	95.511
	b, L mg^−1^	1.306	1.923	0.086	0.179	0.288	0.966	1.954	0.216	0.511
	R^2^	0.993	0.989	0.967	0.998	0.994	1.000	1.000	1.000	0.993
	R_L_, mg L^−1^	0.0013	0.0009	0.0191	0.0092	0.0058	0.0017	0.0009	0.0076	0.0033
Freundlich isotherm	N	6.452	5.682	4.505	4.292	5.952	4.808	5.208	3.521	8.929
	K_F_, mg g^−1^	39.902	35.727	30.437	29.322	41.371	64.269	61.404	48.184	53.654
	R^2^	0.570	0.517	0.576	0.961	1.000	0.657	0.736	0.831	0.552
Temkin isotherm	B, J mol^−1^	0.283	0.246	0.414	0.192	0.256	0.174	0.193	0.107	0.358
	K_T_, L mg^−1^	95.092	27.205	1642.844	5.522	70.061	126.497	165.833	7.579	3340.749
	R^2^	0.538	0.525	0.870	0.981	0.994	0.800	0.774	0.935	0.622
D-R isotherm	Β, mol^2^ KJ^−2^	2.0E-06	8.0E-06	7.0E-06	2.0E-06	2.0E-07	2.0E-07	7.0E-08	8.0E-07	3.0E-07
	Q_m_, mg g^−1^	81.696	84.775	76.876	75.709	79.107	119.426	96.621	145.416	93.878
	E_D-R_, KJ mol^−1^	0.500	0.250	0.267	0.500	1.581	1.581	2.673	0.791	1.291
	R^2^	0.947	0.881	0.787	0.971	0.943	0.803	0.943	0.956	0.576

**Table 5:** Langmuir, Freundlich, Temkin and Dubinin–Radushkevich (D–R) isotherm parameters for adsorption of BB dye onto BL-CNSs.

		BL1-CNSS	BL2-CNSS	BL3-CNSS	BL4-CNSS	BL5-CNSS	BL6-CNSS	BL7-CNSS	BL8-CNSS	BL9-CNSS
Langmiur isotherm	Q_m_, mg g^−1^	24.558	32.000	31.756	2.441	34.990	35.817	70.522	2.506	40.733
	b, L mg^−1^	0.009	0.002	0.009	0.069	0.004	0.024	0.060	0.146	0.149
	R^2^	0.515	0.382	0.977	0.500	0.764	0.845	0.976	0.002	0.989
	R_L_, mg L^−1^	0.154	0.469	0.150	0.024	0.293	0.065	0.027	0.011	0.011
Freundlich isotherm	N	1.636	0.780	0.583	1.171	1.017	1.547	1.275	0.735	2.370
	K_F_, mg g^−1^	1.511	70.909	150.557	4.159	9.183	1.280	3.112	7.698	5.303
	R^2^	0.914	0.930	0.956	0.975	0.949	0.917	0.791	0.917	0.216
Temkin isotherm	B, J.mol^−1^	0.646	0.944	0.351	0.492	0.575	0.381	0.157	0.156	0.345
	K_T_, L.mg^−1^	5.028	12.529	12.055	8.294	9.550	3.028	1.750	7.921	1.994
	R^2^	0.769	0.887	0.951	0.834	0.916	0.791	0.969	0.986	0.372
D–R isotherm	Β, mol^2^ KJ^−2^	1.0E−05	5.0E−05	7.0E−05	2.0E−05	3.0E−05	4.0E−06	3.0E−06	4.0E−05	4.0E−06
	Q_m_, mg g^−1^	8.654	4.135	10.574	8.625	7.587	11.603	42.730	31.507	26.846
	E_D-R_, KJ mol^−1^	0.224	0.100	0.085	0.158	0.129	0.354	0.408	0.112	0.354
	R^2^	0.765	0.891	0.886	0.770	0.855	0.133	0.953	0.967	0.150

From the value of the correlation coefficient factor R^2^ listed in Table 5, it can be noticed that adsorption capacity of BB dye on BLs-CNSs fit better to Freundlich isotherm for all carbon nanostructure except BL7-CNSs and BL9-CNSs that presents high adsorption capacity (40.7 and 70.5 mg/g, respectively); Fig. 5. In agreement with Abdel-Ghani et al. and Arya et al.^26,50^, the adsorption of BB on carbon-based materials fit well to Freundlich isotherm which is valid for heterogeneous surfaces. The large value of n (0.58–2.37) indicates a strong interaction between carbon nanostructure surface and BB dye. With respect to BL7-CNSs and BL9-CNSs samples, they are fit most to the Langmuir isotherm model with R^2^ about 0.98 and lowest R_L_ values (0.011–0.027). The high adsorption capacity of these two samples may be attributed to the surface area or the cationic active sites on the carbon surface. The maximum capacity results are noticed from present work, compared with previously reported findings, given in Table 6.

**Table 6:** Comparison of obtained maximum adsorption capacity (Q) of BB with literatures.

Present work	Q, mg/g	Adsorbent material	Q, mg/g	Ref
BL1-CNSs	24.56	Poly(phenylenediamine) grafted electrospun carbon nanofibers (PPDA-*g*ECNFs)	6–100	Thamer et al., 2019^51^
BL2-CNSs	32.00	Active carbon from *Nigella sativa* waste	14.49	Abdel-Ghani et al., 2017^26^
BL3-CNSs	31.76	Wheat bran grounded powder	6.41	Ata et al., 2012^52^
BL4-CNSs	24.41	Copper oxide/carbon nanocomposites from *Vitex negundo* Linn leaf	9.09	Bhavyasree and Xavier, 2021^53^
BL5-CNSs	34.99	α-Chitin nanoparticles	13.16	Dhananasekaran et al., 2016^54^
BL6-CNSs	35.82	Pine cone activated carbon	49.35	Geçgel and Kolancilar, 2012^55^
BL7-CNSs	70.52	Iron oxide-graphene oxide composite	14.31	Magsino et al., 2020^56^
BL8-CNSs	25.06	Orange peel activated carbon	11.62	Mafra et al., 2013^57^
BL9-CNSs	40.73	Wheat bran	6.41	Ata et al., 2012^52^

##### Adsorption kinetic studies

**Figure 6:**
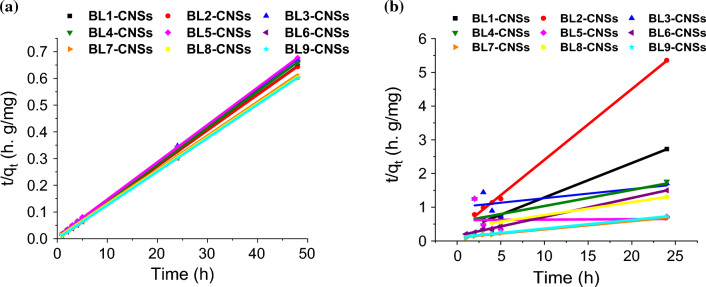
The kinetic fitted curves of MB dye adsorption (**a**) and BB dye adsorption on MAC (**b**) according to pseudo-second-order kinetic model.

The kinetic parameters from the plot of adsorption capacity of MB and BB with the contact time, applying Lagergren first-order, pseudo second order and intraparticle diffusion models are calculated and recorded in Tables 7, 8. Both tables show the values of correlation coefficients (R^2^) of the pseudo second order model (0.999–1) are higher than those calculated from other two models. Moreover, the calculated adsorption efficiencies (Q_eq_) of this model agree with experimental values. Also, the Standard Error of Estimate values (SEE) of the pseudo-second order model are lower than the first order and intraparticle diffusion models. The linearized forms of BLs-CNSs adsorption kinetic model with most fitting curves (pseudo second order) of MB and BB adsorption are displayed on Fig. 6a, b. Fitting the adsorption behavior of both dyes on BLs-CNSs to the pseudo-second-order kinetics model indicates that the adsorption mechanism may be physisorption^51,58^.

**Table 7:** Lagergren first order, pseudo second order and intraparticle diffusion kinetics parameters for MB dye adsorption onto BL-CNSs.

		BL1-CNSS	BL2-CNSS	BL3-CNSS	BL4-CNSS	BL5-CNSS	BL6-CNSS	BL7-CNSS	BL8-CNSS	BL9-CNSS
Lagergren first order	K_1_, h^−1^	0.040	0.018	0.035	0.052	0.090	0.041	0.082	0.053	0.072
	Q_eq_, mg g^−1^	52.055	49.964	52.107	52.599	56.193	46.344	42.470	53.936	43.116
	R^2^	0.942	0.843	0.940	0.946	0.972	0.853	0.954	0.897	0.955
	SEE	0.057	0.028	0.038	0.068	0.075	0.056	0.074	0.061	0.062
Pseudo-second order	K_2_, mg g^−1^	0.039	0.143	0.003	0.013	0.001	0.021	0.006	0.004	0.006
	Q_eq_, mg g^−1^	9.787	4.765	21.993	16.239	62.500	17.775	40.634	26.069	38.895
	R^2^	0.995	0.999	0.959	0.995	0.847	0.997	0.993	1.000	0.991
	SEE	2.440	2.488	2.288	2.475	2.473	0.032	0.020	2.301	0.024
Intraparticle diffusion	K_id_, mg g^−1^ h^−1/2^	6.089	2.921	3.597	3.198	8.619	2.534	6.082	4.826	5.897
	C, mg g^−1^	6.399	2.240	3.082	0.760	7.879	4.471	6.216	4.442	5.027
	R^2^	0.946	0.904	0.957	0.803	0.969	0.823	0.968	0.927	0.975
	SEE	2.215	1.182	1.168	2.454	2.380	1.824	1.725	2.105	1.445

**Table 8:** Lagergren first order, pseudo second order and intraparticle diffusion kinetics parameters for BB dye adsorption onto BL-CNSs.

		BL1-CNSS	BL2-CNSS	BL3-CNSS	BL4-CNSS	BL5-CNSS	BL6-CNSS	BL7-CNSS	BL8-CNSS	BL9-CNSS
Lagergren first order	K_1_, h^−1^	0.040	0.018	0.035	0.052	0.090	0.041	0.082	0.053	0.072
	Q_eq_, mg g^−1^	52.055	49.964	52.107	52.599	56.193	46.344	42.470	53.936	43.116
	R^2^	0.942	0.843	0.940	0.946	0.972	0.853	0.954	0.897	0.955
	SEE	0.057	0.028	0.038	0.068	0.075	0.056	0.074	0.061	0.062
Pseudo-second order	K_2_, mg g^−1^	0.039	0.143	0.003	0.013	0.001	0.021	0.006	0.004	0.006
	Q_eq_, mg g^−1^	9.787	4.765	21.993	16.239	62.500	17.775	40.634	26.069	38.895
	R^2^	0.995	0.999	0.959	0.995	0.998	0.997	0.993	1.000	0.991
	SEE	2.440	2.488	2.288	2.475	2.473	0.032	0.020	2.301	0.024
Intraparticle diffusion	K_id_, mg g^−1^ h^−1/2^	6.089	2.921	3.597	3.198	8.619	2.534	6.082	4.826	5.897
	C, mg g^−1^	6.399	2.240	3.082	0.760	7.879	4.471	6.216	4.442	5.027
	R^2^	0.946	0.904	0.957	0.803	0.969	0.823	0.968	0.927	0.975
	SEE	2.215	1.182	1.168	2.454	2.380	1.824	1.725	2.105	1.445

##### Mechanism of dye removal (proposed adsorption mechanism)

It is widely acknowledged that the adsorption mechanism is influenced by the solution environment, the chemical structure, and the nature of the adsorbent. As a result, a variety of interactions, including ion exchange, hydrogen bonding, π-π interactions, and electrostatic interactions, are suggested for the removal of dyes by adsorption. The suggested adsorption mechanism of MB and BB on the surface of BL-CNSs is physisorption involving electrostatic interactions, hydrogen bonding and π–π interactions (pi effect)^52,53,59,60^. The surface charge of carbon nanostructure tends to be negatively charged in the semi-alkaline pH of MB dye that easily interacts with positively charged MB. This proposed that the adsorption of MB onto the introduced adsorbent surface occurred mainly due to the electrostatic interaction of positively N^+^ group on MB dye with negatively charged backbone of CNSs (carboxyl and hydroxyl group from FTIR). On the basis of the CNSs' FTIRs, which were investigated in the following step, the presence of hydroxyl groups and C=C also also proposed hydrogen bonding and π–π interactions as adsorption mechanisms. In semi alkaline pH, negatively charged CNSs surface might have repelled the incoming negatively sulfonate (SO_3_^−^) groups on BB ions resulting low adsorption, so the BB adsorption mechanism is mainly dependent on hydrogen bonding and π–π interactions over the electrostatic interaction.

#### FTIR-ATR spectra of CNSs

**Figure 7:**
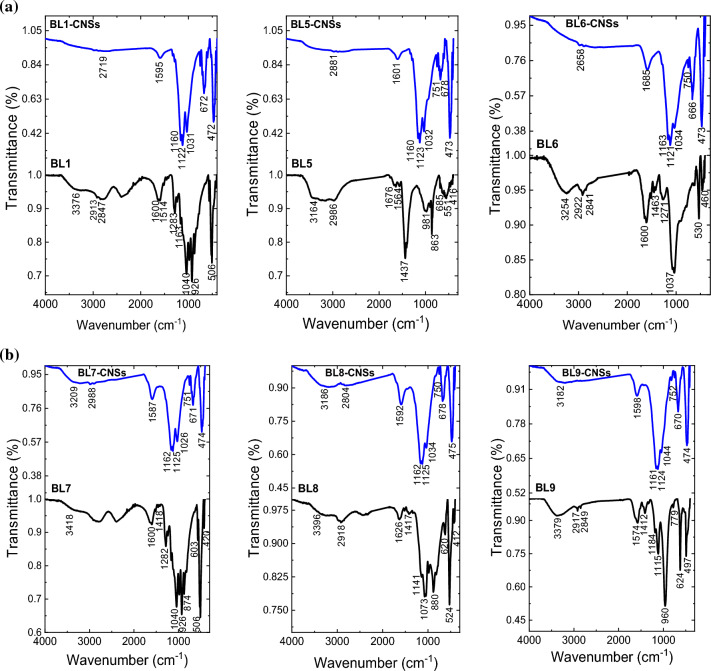
(**a**) FTIR-ATR of selected BL-CNSs prepared from BLs of pulping agents NaOH (BL1-CNSs), M-NaOH-AQ-BH (BL5-CNSs) and KOH/NH_4_OH (BL6-CNSs). (**b**) FTIR-ATR of selected BL-CNSs prepared from BLs of pulping agents, NaOH-Na_2_S, (BL7-CNSs), Na_2_SO_3_/Na_2_CO_3_, (BL8-CNSs) and Na_2_SO_3_ (BL9-CNSs).

Based on the adsorption data and thermal analysis, some BL-CNS samples prepared from BLs of different pulping agents [NaOH (BL1), M-NaOH-AQ-BH (BL5), KOH/NH_4_OH (BL6), NaOH-Na_2_S (BL7), Na_2_SO_3_/Na_2_CO_3_ (BL8), Na_2_SO_3_(BL9)] are selected for further assessment the role of BL pulping on functional groups included the resulting CNSs. These samples are characterized by various adsorption capacities and thermal stabilities. FT-IR spectra of the examined CNSs are displayed in Fig. 7. At about 3200–3400 cm^–1^, broad bands are assigned to hydrogen-bonded –OH stretching vibration of water molecules adsorbed on the carbon surface^54,61^. The peak is around 1580–1680 cm^–1^ design as C=C stretching vibration or C=O group^55,56,62,63^. The sharp band around 1000–1100 cm^–1^ is assigned to O–C–O asymmetric stretching vibration. The sharpness of the ether band is related to the lignin, hemicellulose, or Si–O–Si stretching^57,64^, because the pulping process causes removal of silica from RS, and consequently higher silica content in BLs. Finally, the bands at 600–700 cm^-1^ are assigned to C=C stretching and bending of the benzene ring and the sharp bands at around 400–500 cm^−1^ for Si–O–Si bending. As clear the changes in surface functional groups of CNSs from carbon precursors are the disappearing of the band at 1440 cm^–1^ (CH deformation in CH_3_ or CH_2,_ or phenylpropane skeleton vibrational absorption bands), –OH band broadening, increasing the band related to the Si–O–Si bending indicating the higher silica content. This means that the carbon nanostructure surface has negatively charged carboxyl, hydroxyl and silicate groups that enhance the cationic dye adsorption on these active sites as stated before.

#### Textural characterization of CNSs

**Figure 8:**
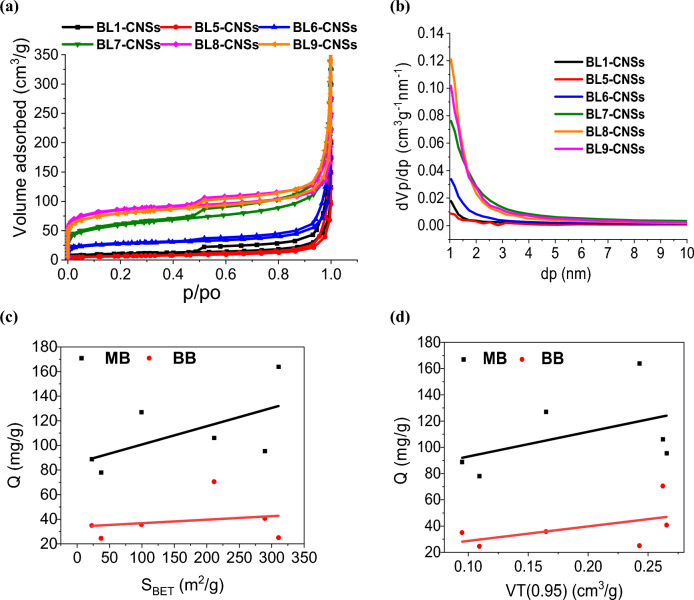
(**a**) N_2_ adsorption–desorption isotherms at 77 K of selected BL-CNSs, (**b**) pore size distributions using NLDFT method. (**c,d**) Maximum adsorption capacity of -CNSs, as a function of their S_BET_ and V_T._

The pore characteristic parameters are important for evaluating porous adsorption materials, Fig. 8a, b. shows the N_2_ adsorption–desorption isotherms and pore size distribution (PSD) that are often used to calculate pore characteristic parameters of the porous materials (Table 9). As clear, the pore characteristic parameters of the pore are greatly affected by the type of pulping process. Based on IUPAC classification, the corresponding results in Fig. 8a and Table 9 exhibits hybrid Type I–IV isotherms with H4 hysteresis^58,59,65,66^. At low relative pressures (0.01–0.1), a sharp increase of the adsorbed nitrogen that indicates microporous structure, however at hysteresis loop occurs between P/P0 = 0.45–0.99 with a slight increase of the adsorbed nitrogen (plateau form) indicates a mesoporous structure. The foregoing results indicate that CNSs material is composed of a mixture of micropores and mesopores.

**Table 9:** The pore structure parameters of selected BL-CNSs.

Code	Carbon yield (C), %	S_BET_, m^2^/g	C × S_BET_, m^2^/g	V_T (0.95)_, cm^3^/g	Pore diameter, nm	S_meso_ (_BJH)_, m^2^/	V_meso (BJH)_, cm^3^/g	d_p peak,_ nm	S_micro (t-plot)_, m^2^/g	V_micro (t-plot)_, cm^3^/g	S_micro_/S_T_, %
BL1-CNSs	55.59	37.02	20.58	0.109	11.810	47.85	0.159	1.050	36.52	0.049	98.65
BL5-CNSs	64.95	22.66	14.72	0.095	16.744	31.62	0.124	1.045	21.76	0.032	96.05
BL6-CNSs	38.96	99.23	38.66	0.165	6.646	94.54	0.225	1.045	63.66	0.008	64.15
BL7-CNSs	17.16	211.28	36.25	0.262	4.967	243.99	0.328	1.045	109.3	0.012	51.73
BL8-CNSs	17.15	310.59	53.27	0.243	3.127	274.10	0.239	1.015	59.80	0.093	19.25
BL9-CNSs	19.27	289.53	55.78	0.266	3.668	261.85	0.298	1.045	74.10	0.078	25.59

It can be noticed that the maximum BET surface areas obtained from the black liquors of neutral and acidic sulfite pulping process (BL8-CNSs and BL9-CNSs; 310.6 and 289.5 m^2^/g, respectively), followed by BL of kraft pulping (BL7-CNSs; 211.3 m^2^/g). However, the remaining surface areas for soda, soda additive and KOH-NH_4_OH BL-CNSs (BL1, BL5 and BL6 CNSs) ranged from 22.7 to 99.2 m^2^/g. Similarly, the total pore volume (V_T_) of BL7-BL9 CNSs is greater than (BL1, BL5 and BL6-CNSs). Whereas the V_T_ of BL7-BL9 CNSs ranges from 0.24 to 0.266 cm^3^/g and the BL1, BL5 and BL6-CNSs have 0.109, 0.095 and 0.165 cm^3^/g. This indicates that the Sulphur-containing BLs are able to produce CNSs with higher surface area and total pore volume as compared with nitrogen-containing BLs. The average pore diameter of all samples ranges from 3.13 to 16.74 nm, with an indirect relationship between the S_BET_ and the average pore diameter.

Figure 8c shows that the performances of MB and BB adsorption on BL-CNSs are almost linearly proportional to S_BET_. So, the suggested reasons of high adsorption capacity of iodine (BL6-CNSs) are the moderate specific surface area (99.2 m^2^/g) and the high micro-surface area (64.2%). While for the high MB cationic adsorption for (BL8-CNSs) is related to the highest S_BET_ (310.6 m^2^/g) and lowest micro-surface area (19.25%) i.e., many mesoporous suitable for the adsorption of macromolecules of MB. The highest BB anionic adsorption for (BL7-CNSs) is often related to the slightly high S_BET_ (211.3 m^2^/g) and highest total pore volume 0.26 cm^3^/g (Fig. 8d), as well as the low negatively active function groups (lowest band intensity of ether linkage at 1000–1100 cm^–1^); Fig. 7. Based on the textural characterization of BL-CNSs, it can be recommended that the by-products of neutral sulfite and Kraft pulping are the best for producing highly efficient BL-CNSs. This behavior is similar to those produced from literature reported lignin and other rice straw by-products, using physical and chemical activation approaches^57,60,64,67^.

#### Scanning electron microscope (SEM)

**Figure 9:**
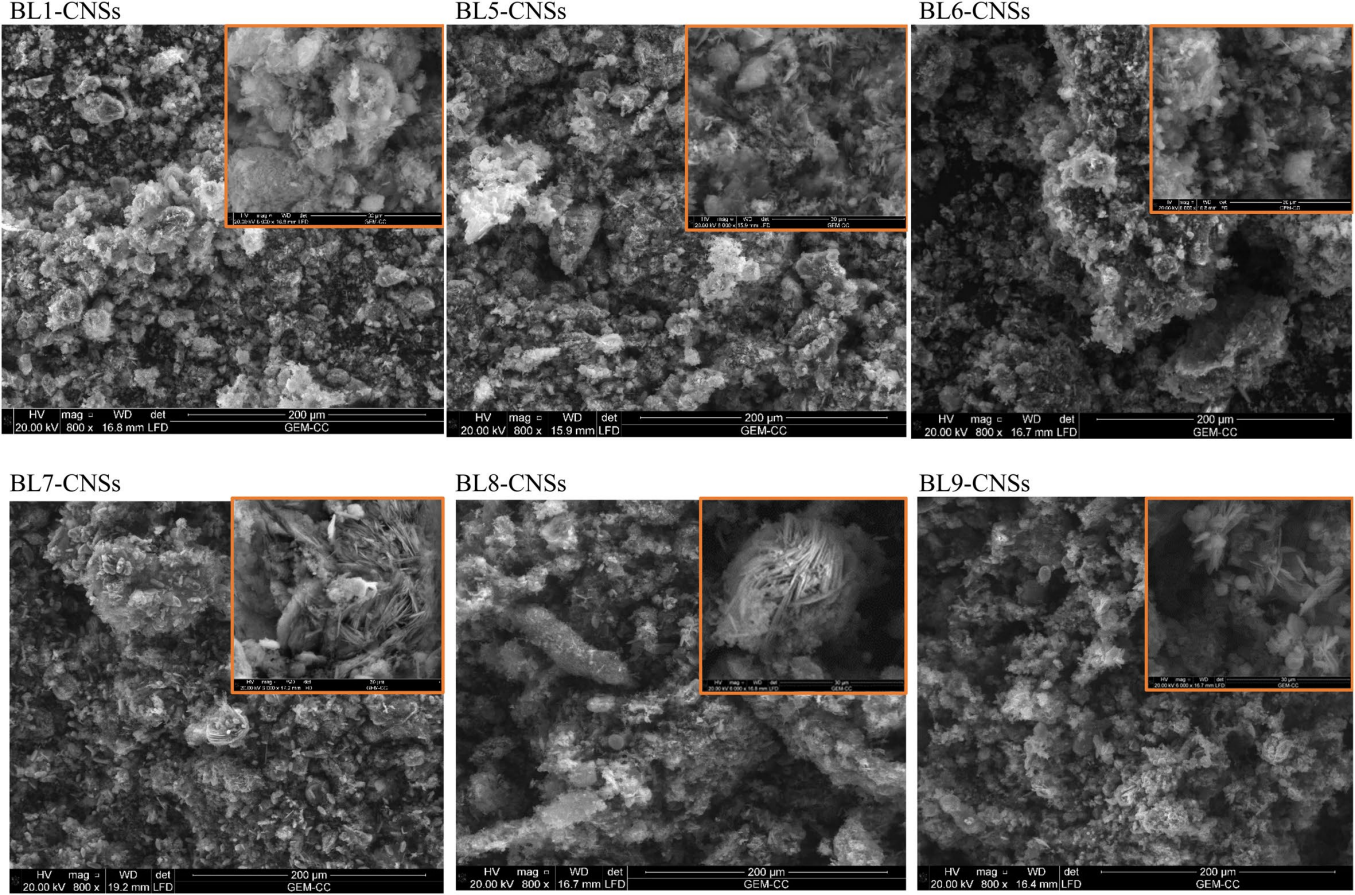
SEM of selected BL-CNSs.

SEM micrographs and microscopic structure of selected BL-CNSs display on Fig. 9. The morphological structure exhibits an inhomogeneous, rough surface with developed pore structure. The presence of silico-phosphate crystals covers carbon surface and specially with high content for BL1, BL5 and BL7-CNSs (soda, soda-additive and Kraft-BL) owing to the high silica removal (58–65%) during the pulping process. Lower crystals are noticed in case of BL6, BL8 and BL9-CNSs of neutral, acidic sulphite and KOH-NH_4_OH pulping of RS due to the lower silica removal (13–15%). The micrographs display the presence of a mixture of micropores and mesopores as presented from textural characterization. Whereas, A mesopore is characterized by a diameter between 2 and 50 nm (IUPAC), whereas a micropore has a diameter of less than or equal to 2 nm.

## Conclusion

This research was conducted to examine and offer sustainable development for the pulping by-products in production of high-performance carbon adsorbent, which was successful in removing dyes discharged from textile industries. A one-step activation procedure employing phosphoric acid as the activating agent was used to produce black liquor-based carbon nanostructures (BL-CNSs). Depending on the pulping procedure, BL-CNSs can be successful in removing pollutants such iodine, MB, and BB. The produced CNSs from the kraft and neutral pulping processes showed the highest adsorption capacities of MB and BB dyes (163.9 and 70.5 mg/g, respectively). The pseudo-second-order model was good to describe the adsorption kinetics, and the Freundlich and Langmuir models, in particular, were well-suited to describe the adsorption equilibrium of the BB and the MB adsorption. The MB and BB dyes were adsorbed on BL-CNSs by a process called physisorption, which involves electrostatic interactions, hydrogen bonds, and π–π interactions. The optimum-prepared adsorbent which prepared from BL of neutral pulping (BL8-CNSs), where it had maximum MB adsorption capacity (~ 164 mg/g), BET surface area of 310.6 m^2^/g, total pore volume 0.243 cm^3^/g and average pore diameter of 3.13 nm.

## Data Availability

All data generated or analyzed during this study are included in this published article.
